# Transient clinical improvement and immune modulation following recombinant Zoster vaccination in a patient with ME/CFS

**DOI:** 10.1016/j.bbih.2026.101388

**Published:** 2026-10-13

**Authors:** AmirAli Bigleri, Emmanuel T. Alimo, Amal Abouda, Mohammed Osman, Jan Willem Cohen Tervaert, Shokrollah Elahi

**Affiliations:** a Mike Petryk School of Dentistry, Division of Foundational Sciences, University of Alberta, Edmonton, AB, T6G 2E1, Canada; b Department of Medicine, Division of Rheumatology, University of Alberta, Edmonton, AB, T6G 2E1, Canada; c Li Ka Shing Institute of Virology, University of Alberta, Edmonton, AB, T6G 2E1, Canada; d Women and Children Health Research Institute, University of Alberta, Edmonton, AB, T6G 2E1, Canada; e Cancer Research Institute of Northern Alberta, University of Alberta, Edmonton, AB, T6G 2E1, Canada; f Glycomics Institute of Alberta, University of Alberta, Edmonton, AB, T6G 2E1, Canada; g Alberta Transplant Institute, Faculty of Medicine and Dentistry, University of Alberta, Edmonton, AB, T6G 2E1, Canada

**Keywords:** Galectin-9, T cell exhaustion, NK cell, Monocytes

## Abstract

**Introduction:**

Myalgic encephalomyelitis/chronic fatigue syndrome (ME/CFS) is a debilitating multisystem disorder associated with immune dysregulation yet lacks definitive biomarkers or effective therapies. Acute immune perturbations may transiently modulate disease manifestations.

**Methods:**

We report the case of a female patient in her 60s with severe, long-standing ME/CFS who experienced changes in symptom following administration of recombinant zoster vaccine (RZV; Shingrix). Peripheral blood mononuclear cells were analyzed pre- and post-vaccination using flow cytometry, with comparison to a female healthy control. Plasma galectin-9 (Gal-9) levels were quantified by ELISA.

**Results:**

Both RZV doses were associated with rapid, transient remission of symptoms, including improved sleep, cognition, pain, mobility, and respiratory function, followed by relapse. Immunologically, pre-vaccination profiling revealed elevated monocytes, increased CD71^+^ erythroid cells, and features of T cell exhaustion marked by Gal-9/PD-1 co-expression and impaired cytokine responses. Post-vaccination, monocyte frequency decreased, Gal-9^+^/PD-1^+^ T cells were reduced, and NK cell degranulation improved, indicating partial restoration of immune function.

**Conclusion:**

This case demonstrates that immune stimulation via RZV may transiently reverse immune dysfunction and improve clinical symptoms in ME/CFS. These findings support a role for reversible immune exhaustion in disease pathogenesis and highlight potential avenues for biomarker development and therapeutic intervention.

## 1 Introduction

Myalgic encephalomyelitis/chronic fatigue syndrome (ME/CFS) is a complex, multi-system disorder characterized by persistent and severe fatigue, post-exertional malaise (PEM), cognitive impairment, unrefreshing sleep, autonomic dysfunction, and other somatic symptoms such as pain resembling fibromyalgia and cognitive dysfunction. It is a profoundly debilitating and is associated with a substantial reduction in quality of life and increased disability (AMaC, 2024; van Eeden et al., 2023).

Despite its clinical severity, the underlying biological mechanisms of ME/CFS remain poorly understood. However, accumulating evidence from multiple studies supports the notion that immune dysregulation is a central feature of the disease. A range of immunological abnormalities has been reported, including altered cytokine profiles, disrupted T-cell activation, and impaired natural killer (NK) cell cytotoxicity, suggesting that both innate and adaptive immune compartments may be dysfunctional (Sotzny et al., 2018; Lacerda et al., 2019). Nonetheless, there are currently no definitive diagnostic biomarkers or effective therapeutic strategies for idiopathic ME/CFS.

The emergence of the COVID-19 pandemic has further underscored the clinical and biological relevance of ME/CFS. A subset of individuals recovering from SARS-CoV-2 infection develop persistent symptoms, commonly referred to as long COVID (LC), that closely resemble those observed in ME/CFS, including chronic fatigue, post-exertional malaise, cognitive dysfunction, and autonomic disturbances (Saito et al., 2024a, 2024b). This overlap has not only strengthened the conceptual link between post-viral syndromes and ME/CFS but has also led to a substantial increase in the number of patients experiencing ME/CFS-like illness. Hence, the post-pandemic landscape presents both an urgent clinical challenge and a unique opportunity to better understand disease mechanisms and identify actionable biomarkers and therapeutic targets.

Collectively, emerging studies in LC patients with ME/CFS-like features reveal a complex and interconnected pathophysiological landscape characterized by widespread immune, hematological, endocrine, and neurobiological dysregulation (Saito et al., 2024b; Eaton-Fitch et al., 2024; Shahbaz et al., 2025a). Persistent low-grade inflammation, impaired interferon signaling, and features of immune exhaustion, including dysfunctional T-cell and NK cell responses with increased expression of inhibitory receptors, suggest chronic antigenic stimulation and ineffective immune resolution (Saito et al., 2024b; Eaton-Fitch et al., 2024). These immune alterations are accompanied by disrupted myeloid cell activity and evidence of impaired erythropoiesis, including expansion of immature erythroid populations CD71^+^ erythroid cells (CECs), which may contribute to systemic hypoxia, redox imbalance, and further immune modulation (Elahi, 2026). In parallel, integrative profiling highlights a coordinated immune–endocrine axis, with sex-specific reductions in key hormones such as testosterone and estradiol correlating with immune dysfunction and symptom severity (Shahbaz et al., 2025b). Transcriptomic analyses further reveal dysregulation of immune, metabolic, and stress-response pathways, alongside unexpected upregulation of neuronal-associated and olfactory receptor genes, pointing to altered neuroimmune communication (Shahbaz et al., 2025a, 2026a). Notably, elevated levels of immune–neuro modulators such as galectin-9 (Gal-9) and artemin link immune exhaustion and chronic inflammation to neurological symptoms, including cognitive impairment (Saito et al., 2024b; Elahi et al., 2024). Together, these findings support a model in which persistent immune perturbation, defective hematopoietic responses, hormonal imbalance, and disrupted neuroimmune crosstalk converge to drive the chronic, multisystem manifestations of LC associated ME/CFS, while also highlighting potential avenues for biomarker development and targeted therapeutic strategies.

Given the proposed role of persistent immune activation and viral reactivation (e.g., the Epstein-Barr virus (EBV), cytomegalovirus (CMV), and herpesviruses) in ME/CFS(13), controlled immune stimuli such as vaccination may offer a unique opportunity to transiently restore homeostasis in these dysregulated pathways.

The recombinant zoster vaccine (RZV; Shingrix) is a subunit vaccine composed of varicella-zoster virus glycoprotein E (gE) combined with the AS01B adjuvant system (Syed, 2018). AS01B contains immunostimulants such as 3-O-desacyl-4′-monophosphoryl lipid A and QS-21, which can activate both innate immune system and enhance adaptive immune responses (Roman et al., 2024). RZV has been shown to induce sustained protective immunity against herpes zoster in adults, with high efficacy in preventing shingles (Baxter et al., 2018).

Although the Shingrix vaccine is widely used to prevent varicella–zoster virus infection, its effects on immune function in individuals with pre-existing immune-mediated and neuroimmune conditions, such as ME/CFS, remain poorly characterized. Emerging observations suggest that acute immune perturbations, arising from infections, immunizations, or other stimuli, may transiently modulate symptom severity in a subset of ME/CFS patients; however, systematic clinical documentation of such effects remains limited.

The findings described in this report emerged as an unexpected clinical observation rather than from a prospectively designed study addressing a predefined research question. The patient, with severe and long-standing idiopathic ME/CFS, contacted our group following a transient remission of symptoms after first dose of RZV vaccination, given our ongoing research on ME/CFS. This prompted an exploratory clinical and immunological assessment aimed at documenting and characterizing the observed phenomenon following the second dose of vaccination. This observation highlights a temporal association between a defined immune stimulus and clinical improvement, offering potential insight into immune-mediated mechanisms underlying ME/CFS and informing potential future therapeutic strategies.

## 2 Materials and methods

**Ethics statement.** This study was approved by the Human Research Ethics Board (HREB) at the University of Alberta (Protocol# Pro00099502 and Protocol # Pro00063463). Written informed consent was obtained from the patient and healthy control (HC). Although the patient received two doses of RZV vaccine, blood sample could not be collected following the first vaccination because she notified us of her transient recovery only after the event had occurred. However, blood samples were collected 2 days before and 4 days after the second vaccination. Two standard doses administered according to the recommended vaccination schedule, with the second dose given within the standard 2–6-month interval after the first dose. The HC was demographically matched (age and sex) and had not received RZV vaccination.

**Blood processing**. Freshly collected peripheral blood was processed within 30 minutes of venipuncture. Plasma samples were collected prior to layering blood onto 15 mL Ficoll Paque gradients (GE Healthcare, Chicago, IL, USA) using standard protocols (Bozorgmehr et al., 2021a; Elahi et al., 2020a). The buffy coat containing peripheral blood mononuclear cells (PBMCs) was collected and washed in RPMI 1640 (Sigma‐Aldrich, Burlington, MA, USA) supplemented with 10% FBS (Sigma‐Aldrich), and 1% penicillin/streptomycin (Sigma‐Aldrich). PBMCs were used immediately for flow cytometry analysis.

**Flow cytometry analysis**. Fluorophore-conjugated antibodies against human cell surface or intracellular markers were purchased from BD Biosciences and Thermo Fisher Scientific. The following antibodies were used: anti-CD3 (SK7), anti-CD4 (RPA-T4), anti-CD8 (RPA-T8), anti-TIM-3 (7D3), anti-CD160 (BY55), anti-2B4 (eBioDM244), anti-TIGIT (MBSA43), anti-PD1 (EH12.1), anti-TNF-*α* (MAB11), anti-IFN-*γ* (45.B3), anti-CD45 (HI30), anti-Galectin-9 (9M1-3), anti-CD16 (B73.1), anti-CD14 (MϕP9), anti-CD56 (B159), anti-CD71 (MA712), anti-CD235a (HIR2), anti-Perforin (dG9), anti-Granzyme-B (GB11), and CD107a (H4A3). Dead cells were excluded using live/dead fixable Aqua stain (Thermo Fisher Scientific cat #L34965). Appropriate FMO (fluorescence minus one) controls were used to define gating strategies and confirm antibody specificity. Cells were stained in 96-well round-bottom plates at 4 °C for 20 minutes using the viability dye, followed by 30-min incubation at 4 °C with surface marker antibodies. For intercellular cytokine staining (ICS), PBMCs were stimulated for 4 hours with anti-CD3 (3 μg/ml) and anti-CD28 (1 μg/ml) in the presence of a Golgi blocker, as previously described (Shahbaz et al., 2021, 2026b). NK cells were subjected to ICS for perforin, granzyme B, and CD107a staining. To minimize variability, identical flow cytometry panels with gating strategies were applied across all samples. A minimum of 100,000 events was acquired per sample according to established protocols (Bozorgmehr et al., 2023, 2024). Compensation controls were prepared using BD CompBeads (BD Biosciences). Following staining, cells were fixed and acquired on an LSRFortessa SORP or Fortessa X-20 (BD Biosciences) and analyzed using FlowJo software (version 10).

**ELISA assay**. Frozen plasma samples at −20 °C were thawed and centrifuged for 15 min at 1500g followed by dilution for Gal-9 concentration using the ELISA kit (R&D, DY, 2045).

## 3 Results

### 3.1 Clinical background and case presentation

The patient is a female in her 60s with a >30-year history of ME/CFS. Her predominant symptoms include severe fatigue, post-exertional malaise, and chronic musculoskeletal pain with associated stiffness, resulting in significant long-term functional impairment. She was previously given a diagnosis of fibromyalgia in the 1990s, which was later withdrawn after lack of sustained response to standard therapies, with symptoms subsequently attributed to ME/CFS and a provisional diagnosis of myofascial pain syndrome. The diagnosis of ME/CFS had been previously established based on comprehensive clinical evaluation in accordance with recognized diagnostic criteria. This included structured assessment using validated ME/CFS diagnostic questionnaires and clinical evaluation consistent with established case definitions (van Eeden et al., 2023; Saito et al., 2024b). No new diagnostic work-up was performed for the present episode, and the diagnosis remained unchanged. No other significant medical comorbidities, relevant family history, or genetic conditions were reported. The patient had previously undergone assessment for chronic viral infections, including EBV serology, without evidence of active or latent infection. However, no formal evaluation for viral reactivation was performed at the time points examined in the present report. The patient has experienced longstanding disability related to ME/CFS, including loss of independent living following clinical deterioration in 2001, and currently requires the use of a cane or walker for ambulation on most days. Prior interventions included pharmacologic treatments for pain and fatigue as well as an intensive aerobic exercise program, after which she experienced clinical worsening and sustained functional decline. The patient reports no known history of COVID-19 infection. She also reports that she received all available COVID-19 vaccinations and underwent SARS-CoV-2 testing on multiple occasions when experiencing respiratory infections, with all test results being negative. Therefore, it is unlikely that she had a symptomatic SARS-CoV-2 infection and subsequently developed LC.

The patient described her condition as severely disabling, with a marked impact on daily functioning and quality of life, and reported transient improvement in mobility and symptom burden during the observed remission period post vaccination. This case was evaluated following a transient remission of symptoms that was temporally associated with administration of the RZV vaccine. At baseline, prior to vaccination, the patient was considered severely affected. She required the use of a cane or walker for ambulation on most days due to significant pain and stiffness. She experienced moderate-to-severe persistent asthma exacerbations triggered by environmental stimuli, including perfumes and chemical exposures. Sleep was highly fragmented, with frequent nocturnal awakenings and non-restorative quality. Diffuse body pain was also a frequent symptom; however, fibromyalgia was not diagnosed, and her musculoskeletal symptoms, including musculotendinous pain and post-exertional stiffness, were attributed to ME/CFS. No defined rheumatologic diagnosis was identified. Based on the patients’ self-report, cognitive endurance was markedly impaired, necessitating frequent caloric intake to sustain mental activity. Additional symptoms included autonomic dysfunction such as orthostatic intolerance, gastrointestinal dysmotility, migraine headaches, and dysregulation of body temperature were also present.

At the time of vaccination, her medication regimen was minimal and included only low-dose metoprolol for management of cardiac ectopy and orthostatic symptoms. No changes to medications occurred before or during the vaccination period.

### 3.2 First RZV vaccination dose

The first dose of RZV was administered intramuscularly on June 17, 2025, at approximately 16:00. Mild systemic symptoms, including generalized body aches, developed later that evening.

Within 24 hours of vaccination, the patient reported a marked and broad improvement across multiple symptom domains. This remission persisted for approximately 4–5 days and was characterized by consolidated and restorative sleep, improved cognition and sustained mental energy, reduced musculoskeletal pain and stiffness, enhanced mobility, absence of asthma symptoms, and complete resolution of migraines. During this period, the patient also required fewer caloric “rescue” snacks to maintain cognitive function (Table 1).

**Table 1:** Combined symptom trajectory following recombinant Zoster Vaccine (Shingrix).

Domain	Pre-shot (baseline)	After first shot (days ∼0–5: remission)	Post remission/relapse after first shot (from ∼Jun 21–23)	After second shot (days ∼0–5: remission)	Post-remission/relapse after second shot (from ∼Oct 2–3)
Sleep	Fragmented, delayed, frequent awakenings	Consolidated, deeper, earlier onset	Fragmented again, worse for ∼48 h then fluctuating	Markedly improved	Mostly reverts; brief good nights reappear intermittently
Cognition/Energy	Brain fog; needs frequent snacks to function	Clearer, better stamina, reduced snack need	Brain fog returns, severe fatigue	Clearer, better stamina, could recover with brief rest	Fluctuating, irritability/low mental energy, severe fatigue
Mobility/Pain	Chronic MSK pain & stiffness; limited mobility	Pain/swelling reduced and mobility improved	Pain/stiffness return, fluctuating thereafter	Pain/swelling reduced, able to walk without aid	Tendinous pain & stiffness; intermittent cane use
Asthma/Resp	Severe to moderate asthma before shot at times	No asthma during remission	Asthma symptoms return	Suppressed entirely (even with scent exposures)	Returns onward
Migraines	Occasional	Absent during core remission	Migraine with relapse onset	Absent during core remission	Migraine began
GI	Chronic dyspepsia tendencies	No symptoms	Nausea, near-syncope esp. after meals	No symptoms	Return of dyspepsia tendencies, low appetite
Autonomic/OI	Severe OI during replace days	Improved exertional tolerance	Severe OI during worst relapse days	Improved exertional tolerance	weakness/twitching re-emerges followed by OI
Temperature	Low baseline (35.5–36.4 °C)	Low-grade “feverish” range (37-38 °C) matching immune response	Variable	Immediate post shot; ∼37-38 °C, sustained for ∼48 h	Around Oct 1: mostly <36.5 °C; occasional spikes back to ∼37 °C for short periods

Abbreviations: Musculoskeletal (MSK), respiratory (Resp), orthostatic intolerance (OI), gastrointestinal (GI).

Between June 21 and 23, the patient experienced a relapse characterized by worsening orthostatic intolerance, headache progressing to migraine, gastrointestinal disturbances, recurrence of asthma symptoms, cardiac irregularities, and increased pain. Symptoms gradually exceeded her pre-vaccination baseline severity. Although, the patient was not continuously monitored through formal clinical assessments during the interval between the two vaccine doses, clinical follow-up during this period was based primarily on patient self-reporting and communication with the study team. The worsening of symptoms following the initial transient improvement was temporary, and after several days her symptoms returned to their pre-vaccination baseline.

### 3.3 Second RZV vaccination dose

The second dose of RZV was administered on September 25, 2025, at approximately 16:00. During the first 48 hours post-vaccination, the patient experienced expected mild systemic symptoms, including low-grade fever (1–1.5 °C increase above her baseline), and body aches.

Despite these transient effects, the patient reported substantial functional improvement within 48 hours of vaccination. Notably, she was able to ambulate without a cane or walker, with normalization of gait observed by September 29. Based on patient's self-report, musculoskeletal pain and joint swelling were reduced, including in the feet, knees, and neck. Asthma symptoms were absent despite typical environmental triggers. Sleep became more consolidated and restorative, with episodes of uninterrupted sleep lasting up to 6 h, an outcome the patient described as highly unusual for her (Table 1).

These improvements persisted across multiple domains for approximately one week, although symptom expression was more variable compared to the response following the first dose. Cognitive endurance improved intermittently; notably, a brief episode of exertion-induced cognitive decline resolved rapidly with food intake, in contrast to her typical prolonged post-exertional recovery.

Beginning on October 2, the patient experienced a relapse characterized by increased fatigue, irritability, exertional intolerance, gastrointestinal symptoms, and recurrent musculotendinous pain and stiffness. She described a reproducible symptom pattern consisting of intense musculotendinous pain and stiffness, followed by upper gastrointestinal autonomic symptoms, and subsequently generalized weakness with muscle twitching as stiffness subsided.

Asthma symptoms recurred, mobility subsequently declined, and symptoms gradually progressed to pre-vaccination.

Clinical outcomes were based primarily on patient-reported symptoms documented during in-person visits and follow-up communications. Following symptom relapse, the patient was contacted weekly and subsequently followed periodically for up to 10 months, during which she continued to experience ME/CFS symptoms. The reliance on patient-reported outcomes, without objective or standardized clinical assessments, represents a limitation of this study.

### 3.4 Immunological differences between the ME/CFS patient and HC and changes following RZV vaccination

To understand potential underlying mechanism associated with transient recovery, we performed immune phenotyping on fresh PBMCs collected from the patient before and after second vaccination and compared them with those from a comparable HC. Before vaccination, the patient exhibited a higher frequency of monocytes, which decreased by approximately 30% following vaccination (Fig. 1A). We further characterized monocyte subsets, including classical (CD14^hi^CD16^-/lo^), intermediate (CD14^hi^CD16^hi^), and non-classical (CD16^hi^CD14-/^lo^) populations. The ME/CFS patient showed a higher proportion of intermediate monocytes compared to the HC (Fig. 1A), a subset commonly associated with pro-inflammatory immune responses (Bozorgmehr et al., 2021b).

**Fig. 1:**
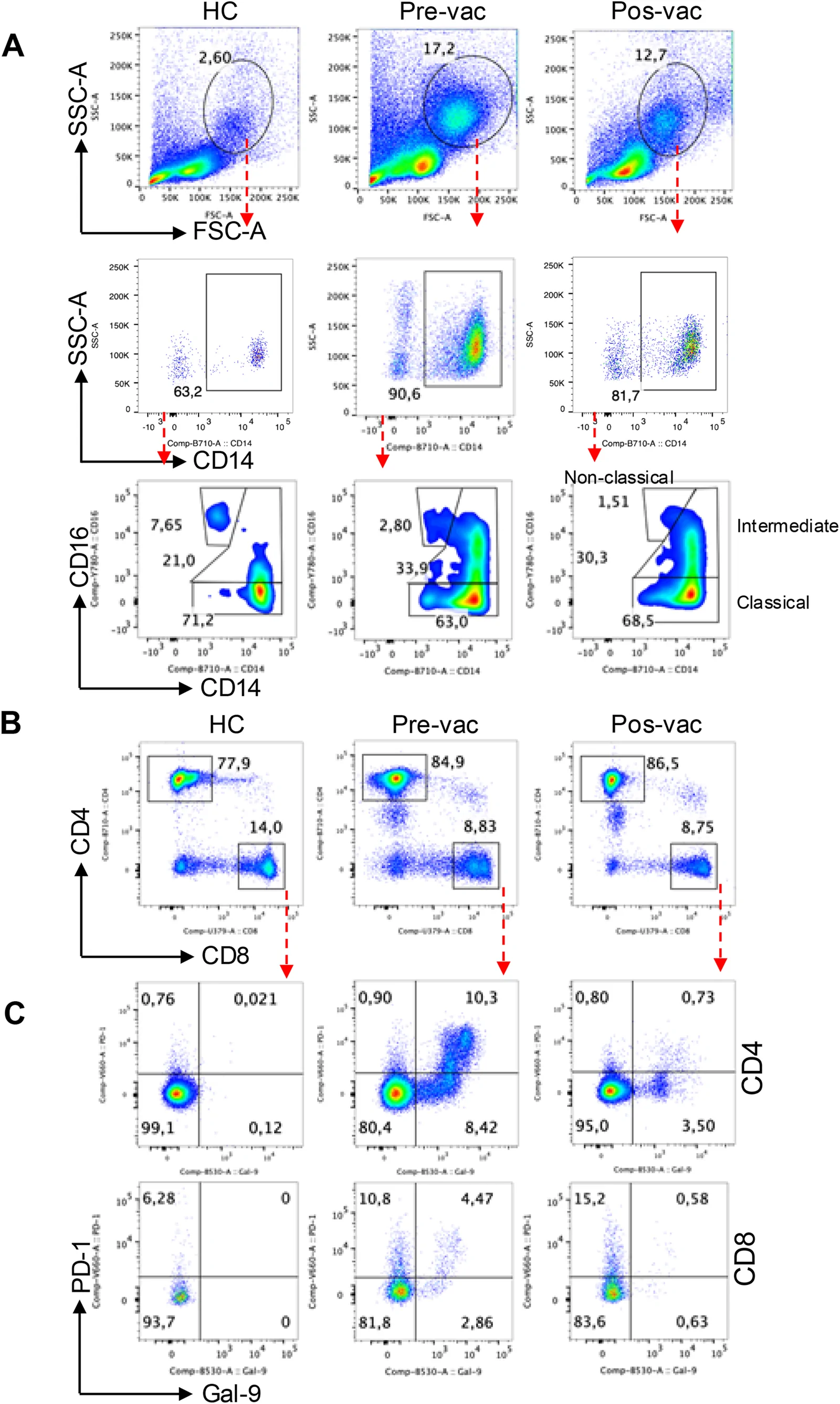
**Increased monocyte frequency and elevated Gal-9+PD-1+ T cells in the ME/CFS patient. A**) Representative flow cytometry plots showing the proportion of monocytes and their subsets among PBMCs from a healthy control (HC), and the ME/CFS patient before vaccination (Pre-vac) and after vaccination (post-vac). **B**) Representative flow cytometry plots depicting the frequency of CD4 and CD8 T cells in HC, Pre-vac, and Post-vac samples. **C**) Representative flow cytometry plots showing co-expression of Gal-9 and PD-1 on CD4 and CD8 T cells from HC, Pre-vac, and Post-vac samples.

Analysis of T cell frequencies revealed no major differences in total T cells pre- and post-vaccination relative to the HC. However, the patient displayed a lower frequency of CD8^+^ T cells compared to the HC (Fig. 1B).

To assess T cell phenotype, we examined the expression of co-inhibitory receptors. Expression of TIM-3, CD160, and TIGIT remained low both before and after vaccination, whereas CD244 (2B4) expression was consistently elevated and did not change following vaccination. In contrast, Gal-9 expression was higher compared with HC and co-expressed with PD-1 on both CD4^+^ and CD8^+^ T cells in the patient (Fig. 1C). Notably, the frequency of Gal-9^+^ CD4^+^ and CD8^+^ T cells was markedly reduced following vaccination (Fig. 1C).

To evaluate T cell function prior to vaccination, we assessed cytokine production following *in vitro* stimulation with anti-CD3/CD28 for 4 hours. PD-1^−^Gal-9^-^ T cells retained responsiveness to TCR stimulation, whereas Gal-9^+^ and Gal-9^+^PD-1^+^ T cells exhibited impaired cytokine production (Fig. 2A). However, such functional assays were not performed after vaccination due to the negligible frequency of Gal-9+ T cells.

**Fig. 2:**
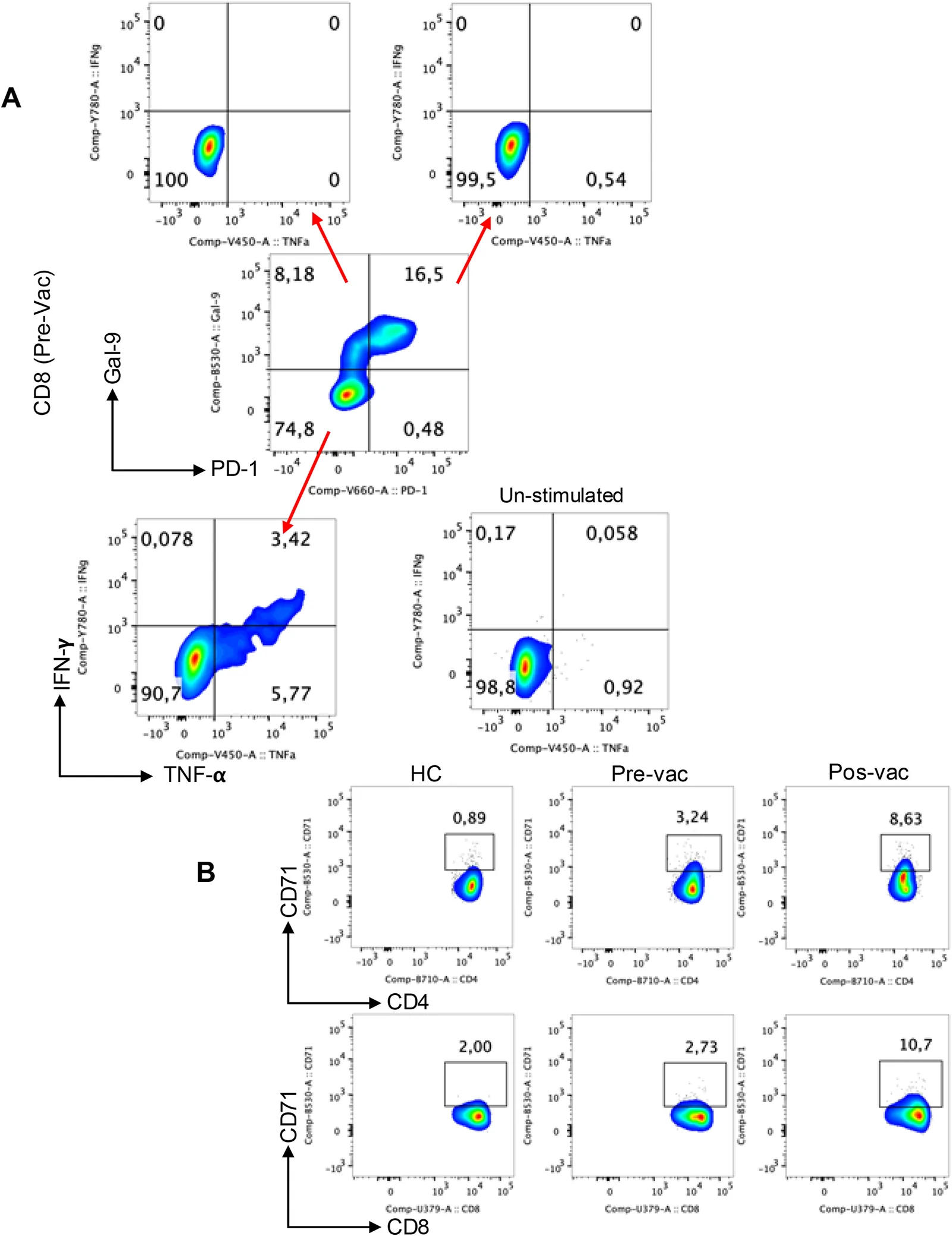
**Functional impairment of PD-1+Gal-9+ expressing CD8 T cells and modest T cell activation following vaccination. A**) Representative flow cytometry plots showing intracellular expression of IFN-γ and TNF-α in CD8 T cells from the ME/CFS patient (Pre-vac) following for 4-h *in vitro* stimulation with anti-CD3/CD28 antibodies. **B**) Representative flow cytometry plots showing the frequency of CD71-expressing T cells in the HC, Pre-vac and Pos-vac samples.

Moreover, we quantified the expression CD71 on both CD4 and CD8 T cells, our observations showed a slight increase in its expression level, which indicates increased T cell activation post vaccination.

NK cell frequency remained unchanged; however, prior to vaccination, NK cells exhibited high intracellular levels of granzyme B (GzmB) and perforin with minimal degranulation capacity (CD107a) (Fig. 3A). Following vaccination, NK cells showed increased degranulation accompanied by reduced intracellular cytolytic content (Fig. 3A).

**Fig. 3:**
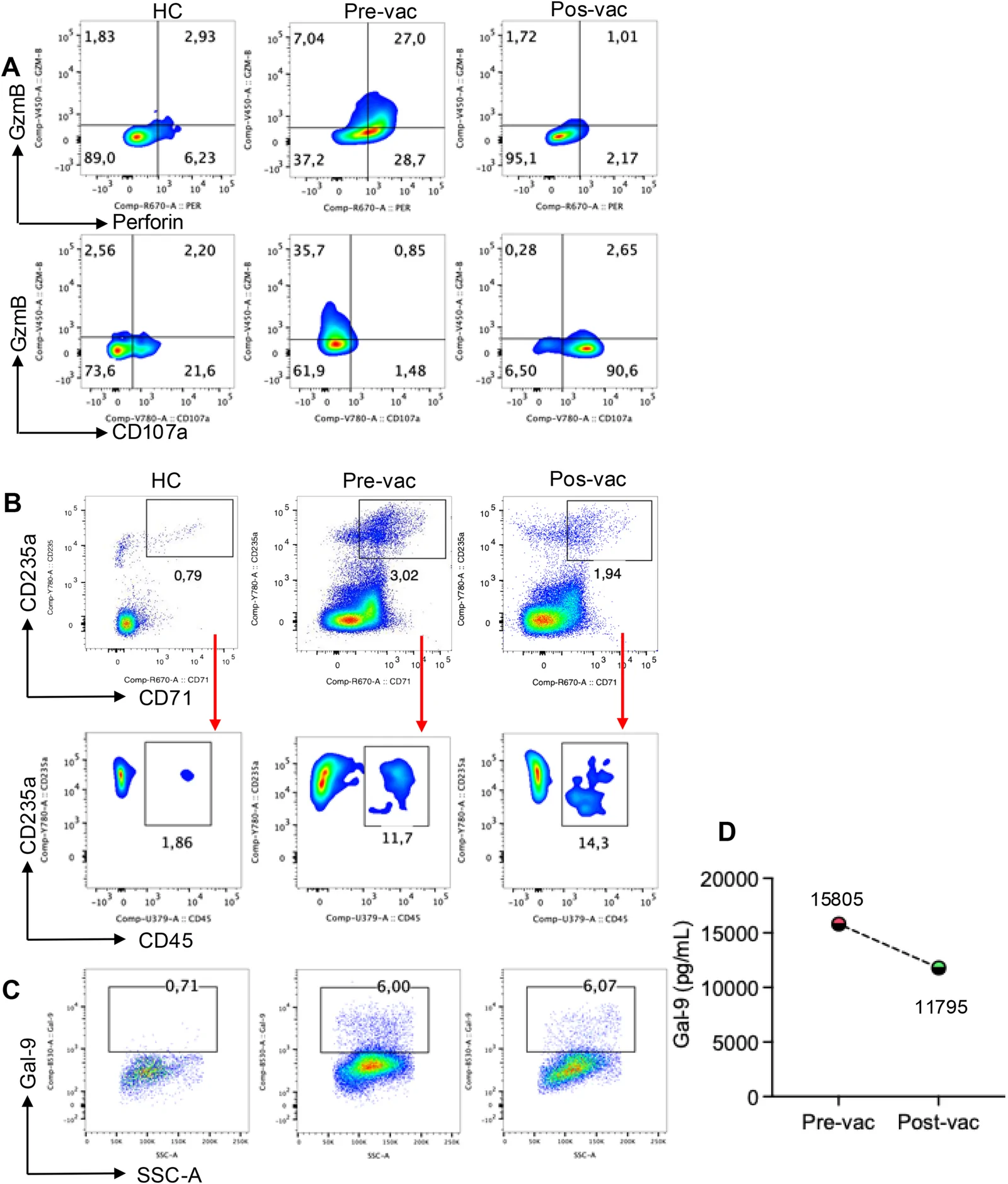
**Impaired NK cell function and increased frequency of CD71^+^ erythroid cells (CECs) in the ME/CSF patient. A**) Representative flow cytometry plots showing intracellular expression of granzyme B (GzmB) and perforin, as well as GzmB and CD107a (degranulation marker) in NK cells from a HC, Pre-Vac, and Post-vac samples. **B**) Representative plots of the frequency of CECs and their CD45^+^ subsets in PBMCs from a HC, Pre-vac, and Post-vac samples. **C)** Representative flow cytometry plots showing the frequency of Galectin-9 (Gal-9)- expressing monocytes in HC, Pre-vac, and Post-vac samples.

Given the reported expansion of CECs in LC patients with ME/CFS(6), we assessed their frequency in this idiopathic ME/CFS patient before and after vaccination. The patient showed an increased frequency of circulating CECs compared to the HC (Fig. 3B). Notably, the proportion of CECs was reduced post vaccination (Fig. 2B). Additionally, we observed a higher proportion of CD45^+^ CECs in the patient both before and after vaccination compared to the HC (Fig. 2B). CD45^+^ CEC are erythroid progenitor cells with more pronounced immunomodulatory properties, and their increased abundance suggest stress erythropoiesis (Elahi, 2026; Elahi et al., 2020b).

Finally, we assessed Gal-9 expression on monocytes and observed higher surface expression in the patient (Fig. 3C). While Gal-9 expression on T cells was reduced following vaccination (Fig. 1B), it remained relatively stable on monocytes (∼6%) both before and after vaccination (Fig. 3C). Consistent with these findings, plasma levels of Gal-9 were elevated and declined following vaccination (Fig. 3D). We have previously shown that a Gal-9 cut-off value > 2779 pg/mL serves as a diagnostic marker in LC patients with ME/CFS, achieving a sensitivity of 78.5% and specificity of 100% (Elahi et al., 2024). The current observation suggests that Gal-9 may also be elevated in idiopathic ME/CFS, indicating its potential broader relevance as a biomarker across ME/CFS subtypes.

Taken together, before vaccination, the patient exhibited several immunological abnormalities consistent with immune dysregulation: increased total and intermediate monocytes, elevated CECs, increased circulating Gal-9, and enrichment of Gal-9^+^PD-1^+^ T cells with impaired cytokine production. NK cells contained high intracellular levels of GzmB and perforin but showed limited degranulation, suggesting impaired cytotoxic function.

Following the second RZV vaccination, monocyte frequency and circulating Gal-9 levels decreased, while Gal-9^+^ and Gal-9^+^PD-1^+^ T-cell frequencies were markedly reduced. CD71 expression on T cells increased, suggesting enhanced activation. NK-cell degranulation improved and was accompanied by reduced intracellular GzmB and perforin, consistent with cytolytic-granule release. In contrast, NK-cell and CECs frequencies remained relatively unchanged. Collectively, these findings suggest a transient shift from an exhaustion-like, functionally impaired immune state toward greater immune activation and effector function, although conclusions are limited by the single-patient design and absence of longitudinal immunological assessment.

## 4 Discussion

In this study, we describe a female patient with long-standing, severe idiopathic ME/CFS who exhibited a rapid, reproducible, and transient remission of symptoms following administration of the RZV vaccine. This clinical response, observed after both vaccine doses, was characterized by broad improvements across multiple domains, including sleep quality, cognitive function, pain, mobility, and respiratory symptoms, followed by relapse to baseline or worse. The reproducibility and temporal association with vaccination suggest that acute immune stimulation may transiently modulate disease activity in ME/CFS.

Our immunophenotyping data provide insight into potential mechanisms underlying this transient improvement. Prior to vaccination, the patient exhibited features consistent with immune dysregulation, including elevated monocyte frequency, increased circulating CECs, and a T cell phenotype suggestive of functional exhaustion, characterized by elevated Gal-9 and PD-1 co-expression and impaired cytokine responsiveness. In addition, NK cells displayed a discordant phenotype, with high intracellular cytolytic content but reduced degranulation capacity, indicative of impaired effector function. Collectively, these findings are consistent with previous reports describing immune exhaustion, dysfunctional cytotoxic responses, and altered hematopoiesis in ME/CFS and LC (Saito et al., 2024b; Hardcastle et al., 2015; Jason et al., 2014).

Following vaccination, we observed changes in immunophenotypic profiles. Monocyte frequency decreased, Gal-9/PD-1 expression on T cells was reduced, and NK cell degranulation capacity improved. These changes were accompanied by an increase in CD71 expression on T cells, suggesting enhanced activation (Motamedi et al., 2016). Importantly, the reduction in Gal-9^+^ and Gal-9^+^PD-1^+^ T cells, populations previously associated with impaired cytokine production (Okoye et al., 2020; Shahbaz et al., 2020), may reflect a shift in T cell phenotypic composition following vaccination. Given that Gal-9 is a key ligand for TIM-3 and is implicated in T cell exhaustion and immune suppression (Elahi et al., 2011, 2012; Elahi and Horton, 2012), its downregulation may represent a central mechanism linking immune reactivation to clinical improvement. Notably, soluble Gal-9 was markedly elevated in this patient, consistent with previous reports in LC patients meeting ME/CSF criteria (Saito et al., 2024b; Elahi et al., 2024). Although Gal-9 can be produced by a variety of activated immune and non-immune cells (Merani et al., 2015), emerging evidence suggest that activated neutrophils may serve as a significant source of circulating Gal-9 in inflammatory conditions (Dunsmore et al., 2021; Perez Rosero et al., 2021). Whether elevated circulating Gal-9 contributes to neuroimmune dysfunction, or whether Gal-9 is also increased within the central nervous system in ME/CFS, as observed in people living with HIV, remains to be determined (Moar et al., 2024; Elahi, 2024).

One plausible explanation for these findings is that the AS01B adjuvant system in RZV induces a strong innate immune activation through pattern recognition receptors (Roman et al., 2024), leading to transient reprogramming of immune responses. This may enhance antigen presentation, cytokine signaling, and effector immune cell function, thereby temporarily overcoming a baseline state of immune exhaustion. In this context, the observed increase in NK cell degranulation and reduction in monocyte expansion may reflect a shift toward a more functional immune profile.

The improvement in cognition, sleep, migraine, pain, and autonomic symptoms may also reflect transient modulation of neuroimmune pathways. Peripheral immune activation induced by RZV could plausibly influence central nervous system function through changes in circulating cytokines and immune mediators, neurovascular signaling, autonomic regulation, or microglial activity (Bastos et al., 2025; Nakatomi et al., 2014). In particular, the post-vaccination reduction in circulating Gal-9 and exhaustion-associated Gal-9^+^PD-1^+^ T cells may be relevant, as persistent immune dysregulation and elevated Gal-9 have been associated with neurological manifestations in post-viral conditions (Elahi et al., 2024; Moar et al., 2024; Elahi, 2024). However, we did not assess cerebrospinal fluid biomarkers, neuroimaging, microglial activation, or other direct measures of central nervous system function (Nakatomi et al., 2014). Therefore, any connection between the observed peripheral immunological changes and neurological improvement remains hypothetical and should be investigated in prospective studies incorporating paired immune and neurological assessments.

Another potential mechanism involves enhanced control of latent viral reactivation. ME/CFS has been associated with reactivation of herpesviruses, including EBV and CMV, which may contribute to chronic antigenic stimulation and immune dysfunction (Shikova et al., 2020). Vaccination-induced immune activation could transiently enhance antiviral immune surveillance, leading to suppression of latent viral activity and subsequent improvement in symptoms. However, as the immune response wanes, viral reactivation or immune dysregulation may recur, contributing to the observed relapse.

The expansion of CECs observed in this patient represents an additional layer of immune regulation. CECs, particularly the CD45^+^ subset, possess potent immunosuppressive properties and have been implicated in modulating T cell and myeloid cell function (Dunsmore et al., 2017, 2018; Elahi and Mashhouri, 2020). Their increased frequency may contribute to systemic immune suppression, redox imbalance, and impaired oxygen delivery. Moreover, the concurrent expansion of CECs and monocytes suggests enhanced myelopoiesis, a feature commonly observed in inflammatory conditions (Elahi, 2022) and in LC patients with ME/CFS (Saito et al., 2024b). It is conceivable that vaccination-induced immune activation transiently counteracts immunosuppression milieu and partially restores immune function, although this requires further investigation.

Importantly, the transient nature of clinical improvement suggests that while immune activation can temporarily restore functional immune balance, it does not fully resolve the underlying pathology. The subsequent relapse, often exceeding baseline severity, may reflect a rebound effect or re-establishment of dysregulated immune networks. This pattern highlights the dynamic and potentially unstable immune state in ME/CFS, in which external perturbations can temporarily shift the system toward or away from homeostasis.

This case has several limitations. As a single-patient observation, the findings may not be generalizable. Although the possibility of biological and assay variability cannot be excluded, we did not observe comparable variability across timepoints in the HC samples processed using the same experimental approach. Nevertheless, the immunologic findings should be interpreted cautiously, as they were based on comparison of two samples collected at different timepoints from a single patient without additional post-relapse sampling. Consequently, some or all of the observed differences may represent normal biological or assay variability. Future studies should aim to systematically evaluate immunological changes in ME/CFS using longitudinal, multi-timepoint sampling across larger cohorts.

Additionally, other factors, including environmental or behavioral variables, cannot be fully excluded. Nonetheless, the reproducibility of the subjective patient response following two independent vaccine exposures strengthens the biological plausibility of the observed effects. Integrating functional assays of T cell and NK cell activity would help clarify whether phenotypic changes translate into true restoration of immune function. In addition, assessment of latent herpesvirus reactivation in relation to immune perturbations may provide important insight into potential viral-immune interactions driving symptom variability. Controlled studies examining responses to defined immune stimuli, such as vaccination, could further help identify reproducible immunological signatures and potential responder subgroups within ME/CFS.

In conclusion, this case provides evidence that targeted immune stimulation may transiently modulate symptoms and immune function in ME/CFS. The observed association between reduced Gal-9/PD-1 expression, improved NK cell function, and clinical remission supports a model in which reversible immune exhaustion contributes to disease pathogenesis. These findings highlight the potential of leveraging controlled immune perturbations to better understand disease mechanisms and identify novel therapeutic strategies for ME/CFS.

## CRediT authorship contribution statement

**AmirAli Bigleri:** Writing – original draft, Visualization, Investigation, Formal analysis, Data curation. **Emmanuel T. Alimo:** Writing – review & editing, Investigation, Formal analysis, Data curation. **Amal Abouda:** Writing – review & editing, Visualization, Data curation. **Mohammed Osman:** Writing – review & editing, Resources, Data curation. **Jan Willem Cohen Tervaert:** Writing – review & editing, Resources, Data curation. **Shokrollah Elahi:** Writing – review & editing, Writing – original draft, Supervision, Resources, Project administration, Funding acquisition, Conceptualization.

## Ethics declaration

Written informed consent to take part in the study and to publish the article has been obtained from all participants or their legal representatives. The privacy rights of participants have been observed.

This study included organ or tissue donors. This study includes human biological material and consent was obtained by donors, or their next of kin or legal representatives, for use in this study and for publication of the article. The samples used in this research were not sourced from executed prisoners or prisoners of conscience.

This study was performed in compliance with relevant laws, regulatory frameworks and guidelines where the research took place. This study was conducted in accordance with the Human Research Ethics Board (HREB). This study was approved by the Human Research Ethics Board (HREB) at the University of Alberta. (Approval No. Protocol# Pro00099502 and Protocol # Pro00063463)

This research follows the CARE guidelines and the CARE checklist.

## AI-associated usage

During the preparation of this work the author(s) used ChatGPT for grammatical correction in some sections of the manuscript. After using ChatGPT, the author(s) reviewed and edited the content as needed and take full responsibility for the content of the publication.

## Declaration of competing interest

The authors declare the following financial interests/personal relationships which may be considered as potential competing interests: Shokrollah Elahi reports a relationship with Canadian Institutes of Health Research that includes: funding grants. If there are other authors, they declare that they have no known competing financial interests or personal relationships that could have appeared to influence the work reported in this paper.

## Data Availability

Data related to this study are included in main and supplemental figures.
